# Effects of a Low‐FODMAP Diet on Symptoms and Quality of Life in Women With Endometriosis: A Systematic Review and Meta‐Analysis

**DOI:** 10.1111/jhn.70353

**Published:** 2026-10-14

**Authors:** Amanda Alves Rabelo, Júlia Fraga Castro e Oliveira, Janete Rabelo Rios, Yasmim de Souza Nunes, Gabriel Bernardes Yacoub, Carolina Alves Corrêa, Guilherme Santos da Silva, Alessandra Bedin‐Pochini

**Affiliations:** ^1^ Bauru School of Medicine (FMBRU‐USP) University of São Paulo Bauru Brazil; ^2^ Pontifical Catholic University of Minas Gerais (PUC‐MG) Belo Horizonte Brazil; ^3^ University of Itaúna (UIT) Itaúna Brazil; ^4^ Afya School of Medical Sciences of Palmas Palmas Brazil; ^5^ Hospital Israelita Albert Einstein (HIAE) São Paulo Brazil; ^6^ Minas Gerais Medical Sciences Faculty (FCMMG) Belo Horizonte Brazil; ^7^ Federal University of São Paulo (UNIFESP) São Paulo Brazil

**Keywords:** endometriosis, low‐FODMAP diet, meta‐analysis, pain, quality of life

## Abstract

**Introduction:**

Endometriosis is a chronic inflammatory gynaecological disease that significantly impairs quality of life. Dietary strategies such as low‐FODMAP diet have been proposed as adjunctive non‐pharmacological approaches to symptom management. This systematic review and meta‐analysis aim to evaluate the association of a low‐FODMAP diet on quality‐of‐life outcomes in women with endometriosis.

**Methods:**

PubMed, Embase, and Cochrane were searched for randomised and observational studies assessing low‐FODMAP dietary interventions in women with endometriosis. Outcomes were evaluated using the Endometriosis Health Profile‐30 (EHP‐30). Pooled analysis was performed using a random‐effects model.

**Results:**

We included three studies comprising 90 women with a mean age of 34 years. The mean follow‐up duration was 3.5 months. The pooled post‐intervention mean score was 28.45 for pain (95% CI: 20.88 to 36.02; *I*² = 61.8%) and 39.33 for control/helplessness (95% CI: 24.92 to 53.73; *I*² = 81.7%). The corresponding post‐intervention mean scores were 31.87 for emotional well‐being (95% CI: 27.15 to 36.59; *I*² = 0%), 38.94 for social support (95% CI: 33.69 to 44.20; *I*² = 0%), and 43.22 for self‐image (95% CI: 35.94 to 50.49; *I*² = 21.2%).

**Conclusion:**

A low‐FODMAP diet was associated with improvements across multiple quality‐of‐life domains in women with endometriosis. However, these findings should be interpreted with caution and considered hypothesis‐generating, requiring confirmation by larger, well‐designed randomised controlled trials.

## Highlights

- This is the first systematic review and meta‐analysis to evaluate the association of a low‐FODMAP diet on quality‐of‐life outcomes in women with endometriosis
- Pooled analysis demonstrates consistent improvements across multiple Endometriosis Health Profile‐30 (EHP‐30) domains, particularly in pain and emotional well‐being
- Dietary benefits may be highly driven by an Irritable Bowel Syndrome (IBS)‐like phenotype, potentially serving as a relevant strategy for patients with concomitant gastrointestinal symptoms
- Due to a low‐to‐very‐low overall certainty of evidence and high risk of bias, findings must be interpreted as hypothesis‐generating, requiring future well‐designed randomised controlled trials

**Abbreviations**
BMI: Body Mass Index
CI: confidence interval
DASS‐21: Depression Anxiety Stress Scale – 21 Items
EHPQ: Endometriosis Health Profile Questionnaire
EHP‐30: Endometriosis Health Profile‐30
FODMAPs: fermentable oligosaccharides, disaccharides, monosaccharides, and polyols
GIQLI: Gastrointestinal Quality of Life Index
IBS: irritable bowel syndrome
IBS‐VAS: Irritable Bowel Syndrome Visual Analogue Scale
MRI: magnetic resonance imaging
NA: not available
NIH PROMIS GI: National Institutes of Health Patient‐Reported Outcomes Measurement Information System Gastrointestinal Symptoms
PRISMA: preferred reporting items for systematic reviews and meta‐analyses
PROSPERO: international prospective register of systematic reviews
RCT: randomised controlled trial
SD: standard deviation
TVUS: transvaginal ultrasound

## 1 Introduction

Endometriosis is a oestrogen‐driven inflammatory condition marked by existence of tissue resembling the endometrium outside the uterine cavity, impacting around 10% of women in their reproductive years and virtually 50% of those facing infertility [1, 2]. The condition is linked to pelvic discomfort, menstrual pain, and digestive issues like bloating, constipation, and abdominal pain, all of which negatively affect quality of life and everyday activities [3, 4]. The first‐line treatment typically involves hormonal suppression therapy, including combined oral contraceptives, progestins, or gonadotropin‐releasing hormone analogues, aimed to reduce oestrogen‐driven lesion activity and pain [5]. However, these therapies often fail to fully control symptoms and may be limited by its side effects [6].

In light of this, recent studies have suggested that use of a low‐FODMAP diet in women with endometriosis may improve gastrointestinal and pelvic symptoms, including reductions in abdominal discomfort, bloating, and constipation [7, 8, 9]. Low‐FODMAP diets limit short‐chain carbohydrates that are inadequately absorbed in the small intestine and fermented by gut bacteria, resulting in gas production and activation of visceral hypersensitivity, which may be related to abdominal and pelvic pain in those patients [10]. By reducing these triggers, low‐FODMAP interventions may alleviate both gastrointestinal symptoms and the visceral pain amplification that commonly exacerbates endometriosis‐associated discomfort.

Previous literature has explored the role of nutrition in endometriosis, summarising heterogeneous dietary interventions such as anti‐inflammatory diets, micronutrient supplementation, and general nutritional counselling, and demonstrating potential improvements in quality of life, gastrointestinal symptoms, and endometriosis‐related pain [11]. Considering the growing clinical focus on the nutritional treatment of endometriosis and the lack of substantial randomised controlled trials, we aim to perform the first systematic review and meta‐analysis to assess the impact of a low‐FODMAP diet on pain and digestive symptoms in women suffering from endometriosis.

## 2 Methods

This systematic review and meta‐analysis were conducted and reported in accordance with the Cochrane Collaboration and the Preferred Reporting Items for Systematic Reviews and Meta‐Analysis (PRISMA) statement guidelines [12] and the Cochrane Collaboration Handbook for Systematic Reviews of Interventions [13]. The complete PRISMA checklist is available in the Supplementary Appendix (Supporting Information S1: Table S2). On August 18, 2025, the prospective meta‐analysis protocol was registered in the PROSPERO database with registration number CRD420251137751.

### 2.1 Eligibility Criteria

Inclusion in this meta‐analysis was restricted to studies that met all of the following criteria: (1) randomised controlled trials or single‐arm observational studies; (2) adult women with a confirmed diagnosis of endometriosis; (3) evaluation of a low‐FODMAP dietary intervention; (4) full‐text articles available in English; and (5) reporting pre and post‐intervention outcomes using the Endometriosis Health Profile‐30 (EHP‐30). Although other validated instruments for assessing quality of life in endometriosis exist, the EHP‐30 is the most widely used, disease‐specific tool with robust psychometric validation [14], and it is the only instrument consistently reported across eligible studies, allowing standardised quantitative synthesis. Because of this, only studies providing EHP‐30 data were included.

We excluded studies that met any of the following criteria: (1) absence of quantitative outcomes measured with the EHP‐30; (2) mixed populations with chronic pelvic pain of non‐endometriosis etiologies without stratified results; and (3) study designs unsuitable for quantitative synthesis, such as reviews, editorials, case reports, conference abstracts, or case series with fewer than five participants. Detailed inclusion and exclusion criteria for each study are provided in Supporting Material (Supporting Information S1: Tables S3 and S4).

### 2.2 Search Strategy and Data Extraction

We systematically searched PubMed, Embase, and Cochrane Central Register of Controlled Trials databases up to August 2025. The complete search strategy for each database is presented in the Supporting Information S1: Table S1 of the Supplementary Appendix. References of included studies, previous reviews, and meta‐analyses were manually screened to identify additional eligible articles.

Two authors (JRR, GBY) independently screened titles and abstracts, and subsequently assessed full‐text articles for eligibility. Disagreements were resolved through discussion or consultation with a senior reviewer. Data extraction was independently performed by two authors (AAR, GSS) using a predefined form, and all extracted data were cross‐checked by a reviewer (JRR). The extracted data included: (1) study characteristics (authors, year, country, design, sample size); (2) participant characteristics (age, diagnostic criteria for endometriosis, stage of disease); (3) details of the low‐FODMAP intervention (duration); and (4) outcomes (EHP‐30 domain scores before and after intervention).

### 2.3 Endpoints

The study outcomes included overall quality of life, assessed by the total EHP‐30 score following adherence to a low‐FODMAP diet, as well as changes across its individual domains. Quality of life was measured using the EHP‐30, with domain scores transformed to a 0–100 scale. Higher scores indicate a worse quality of life, where a score of 0 represents the best possible health status and 100 represents the worst.

The primary endpoint was the pain domain, while secondary endpoints included control and powerlessness, emotional well‐being, social support, and self‐image. Notably, the pain domain of the EHP‐30 reflects the impact and interference of pain on daily activities, social life, and emotional well‐being, rather than serving as a direct measure of physical pain intensity.

### 2.4 Definitions

The included outcomes were analyzed through the EHP‐30 scale, which is composed of two sections: a core questionnaire with five primary domains applicable to all women with endometriosis and an additional modular section comprising six optional domains that address specific aspects relevant to selected patients [15]. The core domains include: (1) pain, which assesses the impact of endometriosis‐related pain on daily life; (2) control and powerlessness, which reflects feelings of lack of control over the disease and its consequences; (3) emotional well‐being, which evaluates the emotional and psychological effects of endometriosis, such as anxiety, sadness, frustration, social support; and (4) self‐image, which captures how endometriosis affects body image and self‐esteem [16].

The use of Oral Contraceptives was defined as the current or recent (within the last 3 months) use of hormonal therapy, which encompassed various regimens such as combined oral contraceptives, progestin‐only pills, or other hormonal treatments frequently prescribed for management of endometriosis‐related pain and symptoms. In addition, gastrointestinal symptoms were evaluated as a composite outcome, primarily focusing on a cluster of complaints that exhibit high clinical overlap with Irritable Bowel Syndrome. These symptoms include bloating, abdominal pain, diarrhoea, constipation, dyschezia and changes in stool consistency or form.

### 2.5 Quality Assessment

The methodological quality of the studies was independently evaluated by two reviewers (J.F.C.O., Y.S.N.), and disagreements were resolved by consensus. The Cochrane Risk of Bias 2.0 tool (RoB‐2) was used for the included RCTs, which were classified as low, some concerns, or high risk of bias according to the domains of randomisation, deviations from intended interventions, missing outcome data, outcome measurement, and selective reporting. The Newcastle‐Ottawa Scale (NOS) was used for the included observational studies, which score studies from 6 to 7 points as low risk of bias, 4‐5 points as moderate risk of bias, and 0‐3 points as high risk of bias.

The quality of evidence was evaluated using the GRADE approach, which rates evidence quality across domains such as risk of bias, inconsistency, indirectness, imprecision, and publication bias, allowing classification of the overall confidence in the effect estimates.

### 2.6 Statistical Analysis

The pooled mean difference (MD) in EHP‐30 total score and other continuous outcomes was calculated using a random‐effects model (DerSimonian and Laird method) due to anticipated heterogeneity among studies. Results are presented as means and standard deviations (SD) with 95% confidence intervals (CI). Heterogeneity across studies was assessed using Cochran's Q test and Higgins and Thompson's *I* ^2^ statistic, with *I* ^2^ values of 0%–40% representing low heterogeneity, 30%–60% moderate heterogeneity, 50%–90% substantial heterogeneity, and 75%–100% considerable heterogeneity. A leave‐one‐out sensitivity analysis (leave‐one‐out method) was conducted in analyses with moderate or high heterogeneity. All analyses were performed using R software (version 4.3.2; R Foundation for Statistical Computing, Vienna, Austria) with meta and metafor packages. For studies in which numerical data were not reported, the corresponding authors were contacted via e‐mail to obtain the necessary information for inclusion in the meta‐analysis.

Due to the lack of standardised comparative data between groups, the analysis was based solely on post‐intervention mean scores. Consequently, the study functions as an exploratory single‐arm synthesis rather than a traditional, confirmatory meta‐analysis. Therefore, the results do not control for confounding variables like the placebo effect and cannot be used to prove treatment efficacy.

### 2.7 Qualitative Analysis

Additional domains could not be included in the quantitative synthesis because they were reported by only two studies, limiting the statistical power required for the meta‐analysis. Consequently, these outcomes were narratively synthesised. They comprised work life, relationship with children, sexual relationship, interaction with healthcare professionals, treatment experience, and infertility.

## 3 Results

The systematic search identified a total of 1600 studies across the databases. After the removal of duplicates, 1220 records remained for title and abstract screening. Of these, 1200 were excluded for not meeting the inclusion criteria. Among the remaining 20 studies assessed in the full text, 12 investigated other anti‐inflammatory diets, three focused on different pathologies, and two lacked dietary intervention. Ultimately, two prospective cohort studies and one RCT [17, 18, 19] were included in this study, comprising 90 patients (Figure 1).

**Figure 1:**
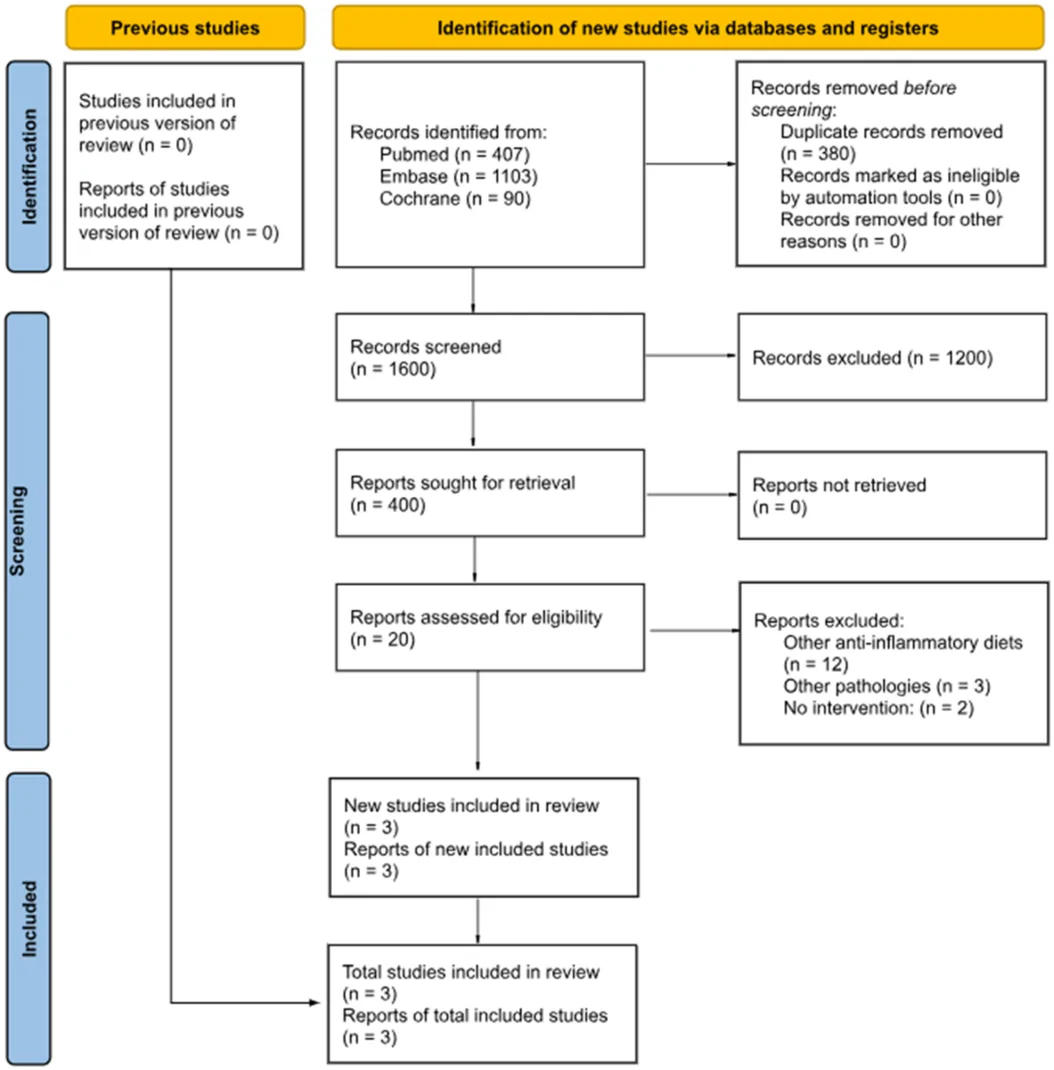
PRISMA 2020 flow diagram for new systematic reviews, which included searches of databases and registers only.

The mean age of participants was 34, ranging from 31 to 37 years. The mean BMI value was 26, ranging from 24.3 to 27 kg/m^2^. The follow‐up durations varied from 1 to 6 months. The use of oral contraceptives ranged from 12.5% to 71% across studies. The proportion of participants reporting gastrointestinal symptoms at baseline ranged from 9% to 58%. Table 1 summarises the baseline characteristics of the included studies (Table 1).

**Table 1:** Baseline characteristics of included studies.

Characteristics	Haaps et. al, 2023 [17]	Keukens et. al, 2025 [18]	Varney et. al, 2025 [19]
Country	Netherlands	Netherlands	Australia
Study Design	Prospective cohort	Prospective cohort	RCT
Intervention group, *n* (%)	22 (54%)	24 (100%)	15 (60%)
Control group, *n* (%)	19	NA	10
Age, y*	36.9	32.5	31
BMI, kg/m^2^ *	26.0	24.3	27
Smoking, *n* (%)	1 (4.5)	10 (55.6)	2 (6)
Educational attainment, *n* (%)	10 (45.5)	9 (37.5)	NA
Current treatment, *n* (%)	16 (72.7)	3	NA
Oral contraceptives, *n* (%)	10 (45.5)	3 (12.5)	25 (71)
Gastrointestinal symptoms *n* (%)	2 (9.1)	14 (58.3)	51.10* (I: 46.8; C: 55.4)
Follow‐up duration	6 months	14 weeks	28 days
Assessment tools	Pain (Visual Analogue Scale, 0–10 cm) and quality of life (EHP‐30: Endometriosis Health Profile‐30).	Constipation (Constipation Scoring System, 0–30), bloating (Groningen DeFeC questionnaire and patient‐reported symptom), and quality of life (EHP‐30).	Gastrointestinal symptoms (IBS‐VAS, NIH PROMIS GI Symptom Scales, stool diary/Bristol Stool Chart), quality of life (GIQLI, EHPQ), and psychological symptoms (DASS‐21).

* mean; C: Control; DASS‐21: Depression Anxiety Stress Scale‐21 Items; EHP‐30: Endometriosis Health Profile‐30; EHPQ: Endometriosis Health Profile Questionnaire; GIQLI: Gastrointestinal Quality of Life Index; I: Intervention; IBS‐VAS: Irritable Bowel Syndrome Visual Analogue Scale; MRI: Magnetic Resonance Imaging; NA: Not Available; NIH PROMIS GI: National Institutes of Health Patient‐Reported Outcomes Measurement Information System Gastrointestinal Symptoms; RCT: Randomised Controlled Trial; TVUS: Transvaginal Ultrasound.

### 3.1 Pooled Analysis

#### 3.1.1 Quantitative Analysis

The pooled mean score for pain among participants was 28.45 (95% CI: 20.88–36.02; *I* ^2^ = 61.8%; Figure 2). The pooled mean score for control and helplessness among participants was 39.33 (95% CI: 24.92–53.73; *I* ^2^ = 81.7%; Figure 3).

**Figure 2:**
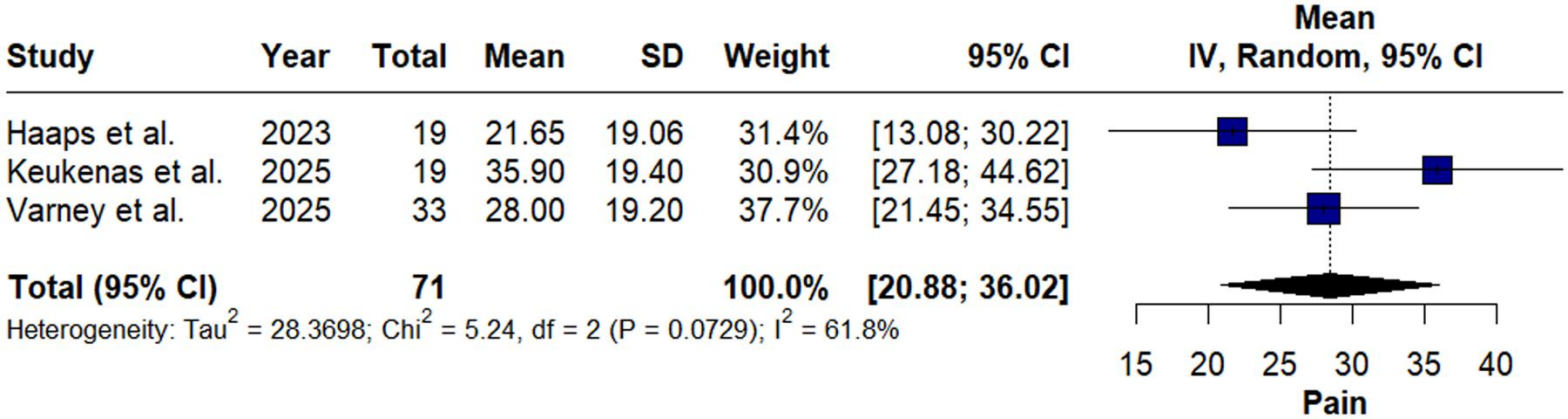
Post‐intervention pooled mean pain domain scores (EHP‐30) in women with endometriosis undergoing low‐FODMAP dietary intervention (mean 28.45; 95% CI: 20.88–36.02).

**Figure 3:**
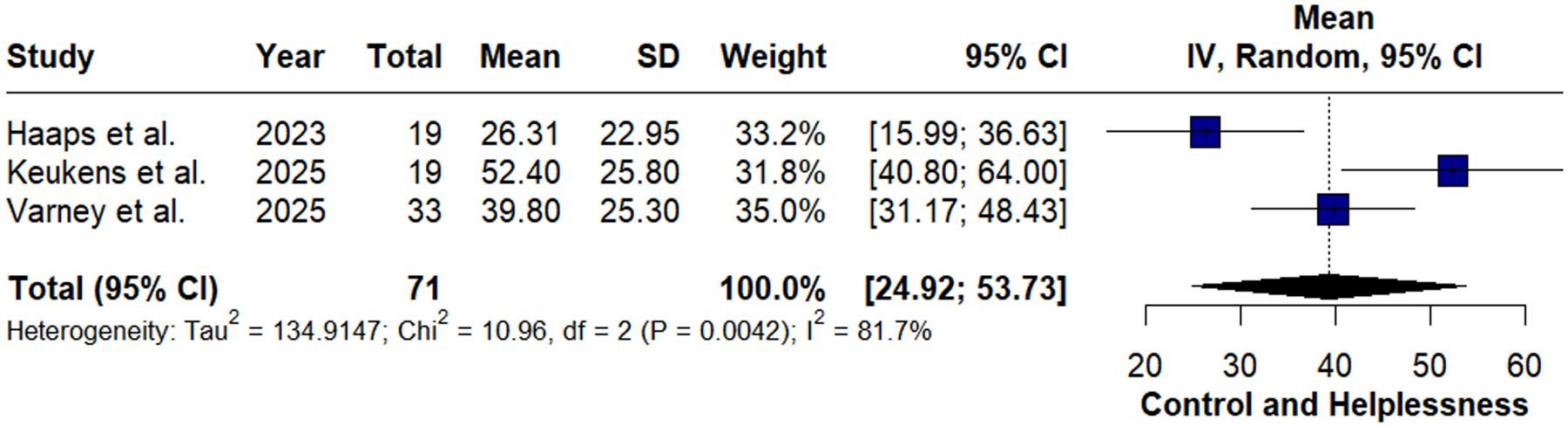
Moderate lack of control and helplessness remained after low‐FODMAP intervention (mean 39.33; 95% CI 24.92–53.73 on EHP‐30).

The pooled mean score for emotional well‐being yielded a pooled mean of 31.87 (95% CI: 27.15–36.59; *I* ^2^ = 0%; Figure 4). Similarly, social support demonstrated a pooled mean of 38.94 (95% CI: 33.69–44.20; *I* ^2^ = 0%; Supporting Information S1: Figure S1).

**Figure 4:**
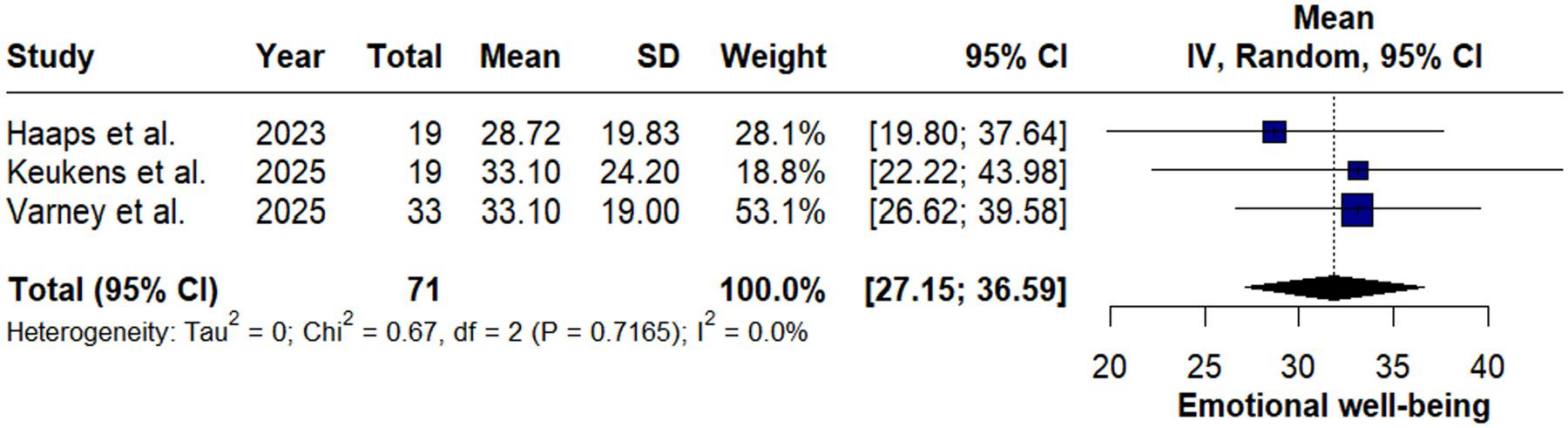
Favourable emotional well‐being was observed after low‐FODMAP diet (mean 31.87; 95% CI: 27.15–36.59 on EHP‐30).

Finally, the pooled mean for self‐image presented a pooled mean of 43.22 (95% CI: 35.94–50.49; *I* ^2^ = 21.2%; Supporting Information S1: Figure S2).

#### 3.1.2 Qualitative Analysis

##### 3.1.2.1 Quality of Life

In the cohort evaluated by Haaps et al., participants exhibited lower mean scores across nearly all quality of life and psychosocial domains when compared with Keukens et al. The most differences were observed in professional life, relationships with children, and particularly sexual relationships (Supporting Information S1: Table S4). Beyond direct comparisons between Haaps et al. and Keukens et al., baseline EHP‐23 scores reported by Varney et al. consistently reflected a moderate‐to‐severe impact on quality of life. Work‐related scores ranged from approximately 40 to 60, sexual relationship scores frequently exceeded 50, and infertility‐related concerns represented the highest burden.

##### 3.1.2.2 Professional Life

Scores related to professional life differed between studies. Haaps et al. reported lower mean and standard deviation scores (14 ± 16.9), whereas Keukens et al. demonstrated nearly twice the mean value (26.2 ± 25.5). This pattern indicates that work‐related limitations may have been more prominently perceived in the Keukens cohort.

##### 3.1.2.3 Relationship With Children

Impairment in the relationship with children was more pronounced in the study by Keukens et al., which reported a median score of 27.1 (IQR 40.6). In contrast, Haaps et al. reported no impairment across follow‐up time points, with median scores of 0.0 at both 3 months (IQR 21.9) and 6 months (IQR 9.4). These findings suggest a greater perceived impact on the relationship with children among participants in the Keukens cohort compared with those in the Haaps study.

##### 3.1.2.4 Sexual Relationships

Sexual relationships demonstrated the most pronounced divergence between studies. Participants in Keukens et al. reported substantially greater impairment (mean ± SD: 54.0 ± 31.0) compared with those in Haaps et al. (30.3 ± 26.9). This difference highlights the substantial and persistent impact of endometriosis‐related symptoms on sexual function, with a notably greater burden observed in the Keukens cohort.

##### 3.1.2.5 Interactions With Healthcare Professionals

In contrast to other qualitative domains, interactions with healthcare professionals were associated with higher mean scores in the study by Haaps et al. (17.5 ± 12.0) compared with Keukens et al. (12.5 ± 43.8). However, the wide variability observed in the Keukens cohort suggests substantial heterogeneity in patient experiences. These findings may indicate a relatively more favourable perception of healthcare interactions among participants in the Haaps cohort, although this interpretation should be made with caution given the variability in reported scores.

##### 3.1.2.6 Treatment‐Related Perceptions

Treatment‐related perceptions were consistent across studies. Both Haaps et al. (41.66 ± 22.9) and Keukens et al. (41.7 ± 27.7) reported nearly identical mean scores, suggesting that perceptions of treatment burden, adherence, and perceived benefit were comparable despite differences in study populations and settings.

##### 3.1.2.7 Infertility Concerns

Infertility concerns exhibited the highest mean scores across all qualitative domains. The greatest burden was reported in the study by Keukens et al. (62.5 ± 43.8), followed by Haaps et al. (mean 56.3; SD not reported). These findings highlight infertility as a prominent and persistent concern among individuals with endometriosis across independent cohorts, although direct comparability is limited by the absence of dispersion measures in one of the studies.

### 3.2 Risk of Bias

#### 3.2.1 NOS Risk of Bias Assessment for Observational Studies

Across the observational studies, one study scored 5/9 on the NOS (fair quality) due to limited comparability between groups and incomplete adjustment for potential confounding factors, and another study scored 3/9 (poor quality) due to deficiencies in participant selection, lack of a clearly defined non‐exposed cohort, and inadequate follow‐up. In Haaps et al. (2023), criteria were fulfilled in the selection and outcome domains. In Keukens et al. (2025), some items in the selection and follow‐up domains were not fulfilled. With low comparability in both studies, confounding is the dominant issue; observed associations may reflect baseline imbalances rather than true effects (Supporting Information S1: Table S5).

#### 3.2.2 RoB 2 Risk of Bias Assessment for Randomised Studies

The remaining study showed an overall high risk of bias due to deviations from intended interventions and missing outcome data, along with some concerns about selective reporting (Supplementary Table 6 *)*.

### 3.3 Leave‐One‐Out Sensitivity Analysis

To assess the robustness of the findings, a leave‐one‐out sensitivity analysis was conducted for pain scores (Supporting Information S1: Figure S3). The overall estimate remained consistent (pooled mean: 28.45; 95% CI 20.88–36.02), with pooled means ranging from 25.45 to 31.40 after sequential exclusion of individual studies. Although no single study disproportionately influenced the pooled mean, the heterogeneity was sensitive to the exclusion of Varney et al. (2025), with *I* ^2^ ranging from 24.9% to 80.8%, highlighting that study‐specific effects contributed notably to between‐study variability [19].

### 3.4 GRADE Assessment

The certainty of evidence for outcomes assessing the effects of a low‐FODMAP diet in women with endometriosis was low to very low (Supporting Information S1: Table S7). This was mainly due to serious risk of bias, substantial inconsistency across studies, and consistent imprecision related to small sample sizes and wide confidence intervals. Although indirectness was not considered serious, publication bias was strongly suspected given the limited number of available studies, further reducing confidence in the effect estimates.

## 4 Discussion

### 4.1 Main Findings and Comparison With Previous Literature

In this systematic review with meta‐analysis, we analyzed post‐intervention outcomes in women through EHP‐30 domains following a low‐FODMAP diet. The main findings from the combined analysis demonstrated that the highest pooled score improvement occurred in the control and powerlessness domain of the EHP‐30, based on within‐group comparisons with baseline. Additionally, consistent improvements were observed in the primary outcome of pain intensity, as well as in emotional well‐being and social support, following the dietary intervention.

Our findings regarding pain‐related quality‐of‐life outcomes align with previous literature. Both Keukens et al. (2025) and Varney et al. (2025) reported substantial improvements after adherence to a low‐FODMAP diet. Although absolute reductions varied across studies, the overall effect sizes were similar to those documented by van Haaps et al. (2023), Keukens et al. (2025), and Varney et al. (2025), all of which documented significant reductions in EHP‐30 pain domains following structured low‐FODMAP protocols. These results suggest that the therapeutic potential for pain improvement is consistent across the available literature, despite differences in study design.

### 4.2 Biological Mechanisms and the IBS‐Endometriosis Overlap

The quality‐of‐life improvements observed in this meta‐analysis closely mimic findings from well‐established low‐FODMAP interventions in patients with Irritable Bowel Syndrome (IBS). As noted by Varney et al. (2025), the symptomatic and QoL responses in women with endometriosis “mimicked those observed during the pivotal feeding study in IBS”, where the reduction of gastrointestinal triggers leads to significant gains in health‐related quality of life.

From a mechanistic perspective, a low‐FODMAP diet reduces fermentable carbohydrate intake, leading to less gas production, intestinal distension, and discomfort. This physiological change triggers less visceral hypersensitivity, a shared mechanism in both conditions that results in lower pain thresholds for internal organs [18]. Given that patients with endometriosis are approximately three times more likely to meet the criteria for IBS compared to the general population [20], the improvements observed in EHP‐30 domains, particularly pain and control/powerlessness, likely reflect a similar pattern of increased sense of control and symptom relief widely described in the IBS literature.

More recently, an umbrella review also highlighted that the relationship between diet and endometriosis remains controversial, with promising but still inconsistent evidence across different dietary strategies [21]. In this context, the low‐FODMAP diet differs from broader anti‐inflammatory dietary approaches because it primarily targets fermentable carbohydrate‐related gastrointestinal symptoms, bloating, intestinal distension, and visceral hypersensitivity [9, 10]. Therefore, its potential benefit in women with endometriosis may be particularly relevant for those with concomitant gastrointestinal symptoms or IBS‐like features, rather than for the broader endometriosis population [20].

### 4.3 Clinical Guidelines and Multimodal Management

These findings support dietary modification as a promising adjunctive strategy within the broader spectrum of lifestyle‐based interventions for endometriosis care. While Kaveh et al. (2025) demonstrated that pharmacological treatment promotes some improvement in overall quality of life, global EHP‐30 scores in their study remained elevated, implying persistent impairment in subjective well‐being despite standard therapy, particularly among women with advanced disease stages [22]. Our meta‐analysis suggests that dietary intervention may offer valuable benefits as an additional, non‐pharmacological strategy.

This perspective aligns with the growing non‐pharmacological and lifestyle‐based strategies in endometriosis care. In this context, a recent meta‐analysis by Xie et al. (2025) synthesised six randomised controlled trials investigating exercise‐based interventions (including yoga, combined training, and relaxation techniques), reporting similar significant improvements in EHP‐30 domains like pain, control, and emotional well‐being [23].

This is further supported by the 2022 ESHRE Endometriosis Guideline, which acknowledges the possible benefits of lifestyle and dietary interventions as part of a holistic approach [24]. Although the guideline states there is currently insufficient high‐quality evidence to make specific recommendations for any nutritional intervention to reduce pain or improve fertility, it actively encourages physicians to discuss healthy lifestyle choices—including a balanced diet—as a non‐drug strategy to manage symptoms and support the continuous psychological well‐being of patients.

Previous reviews on dietary interventions in endometriosis have evaluated heterogeneous strategies, including anti‐inflammatory dietary patterns, Mediterranean‐style diets, antioxidant supplementation, omega‐3 fatty acids, vitamin D, and general nutritional counselling [8, 11]. Overall, these interventions have suggested potential improvements in pain, inflammatory status, gastrointestinal symptoms, and quality of life; however, the available evidence remains limited by small sample sizes, heterogeneous protocols, variable outcome measures, and short follow‐up periods.

### 4.4 Methodological Limitations and Certainty of Evidence

Despite the promising signals, these findings must be interpreted with strict caution in the context of several severe methodological limitations. A central limitation is that the pooled estimates were based primarily on post‐intervention EHP‐30 scores rather than between‐group differences or adjusted treatment effects. As a result, this analysis functions closer to an exploratory single‐arm synthesis rather than a conventional confirmatory treatment‐effect meta‐analysis.

Consequently, the analysis cannot control for placebo effects, expectation bias, reporting bias, regression to the mean, natural symptom fluctuation, or the influence of concurrent therapies. This is particularly relevant since the use of hormonal suppression therapy was highly prevalent across the cohorts, ranging from 12.5% to 71% of participants. Therefore, no definitive causal inference regarding the efficacy of the low‐FODMAP diet can be made from these pooled estimates.

Moreover, the overall evidence base is small and constrained by high risk of bias. Of the three analyzed studies, two were single‐arm prospective observational cohorts [17, 18] and only one was a randomised controlled trial [19]. The two observational studies displayed moderate to poor methodological quality according to the Newcastle‐Ottawa Scale, limited by a lack of comparability, incomplete adjustment for confounders, and flaws in participant selection.

The sole randomised controlled trial was classified as having a high risk of bias due to deviations from intended interventions and missing outcome data. Compounding this, all outcomes were based on subjective, patient‐reported questionnaires (EHP‐30) without objective clinical correlates. Although the instrument is validated and widely used for symptom assessment and quality‐of‐life evaluation in endometriosis, subjective outcomes remain susceptible to placebo effects, expectation bias, and reporting bias, which may influence the interpretation of treatment effects. Accordingly, the GRADE assessment indicated a low to very low certainty of evidence across all outcomes, reducing our confidence in the pooled estimates and precluding definitive conclusions.

### 4.5 Heterogeneity and Population Phenotypes

Significant clinical heterogeneity across the dietary interventions also warrants consideration. The included studies utilised distinct primary outcome measures: Varney et al. (2025) focused on the proportion of responders to overall gastrointestinal symptoms, while Haaps et al. (2023) prioritised general pain reduction, and Keukens et al. (2025) targeted changes in constipation scores.

Intervention duration and timing of outcome assessments varied considerably across studies. Varney et al. evaluated outcomes strictly after a 4‐week elimination phase (Step 1), whereas Keukens et al. measured effects after a minimum of 14 weeks, following the completion of the reintroduction period, and Haaps et al. conducted assessments at 6 months. Finally, delivery protocols ranged from a highly controlled feeding study providing portioned meals to maximise adherence [17, 18, 19] to dietitian‐taught interventions more reflective of real‐world clinical practice [18].

These methodological discrepancies likely contributed to the observed variability in results and limit the ability to define a standardised low‐FODMAP protocol for this population. Intervention duration, protocol intensity, reintroduction phase, dietary supervision, and adherence monitoring all differed, reducing direct comparability across studies and confidence in the pooled estimates.

Participant characteristics can also influence the observed effects. Most of the women included had moderate to severe symptoms, potentially limiting a comprehensive analysis to women with milder disease. Crucially, the baseline burden of gastrointestinal (GI) symptoms varied across studies. Keukens et al. (2025) and Varney et al. (2025) selectively recruited participants with debilitating GI symptoms (with 58.3% of the Keukens cohort meeting Rome III criteria for IBS).

In contrast, in the study by Haaps et al. (2023), only 9.1% of the low‐FODMAP group had formal gastrointestinal or urinary diagnoses at baseline. Because of this selective recruitment, the observed improvements may reflect a response to FODMAP restriction in an IBS‐like phenotype rather than a direct effect on endometriosis‐specific mechanisms. Thus, the findings may be more applicable to selected women with endometriosis and concomitant gastrointestinal symptoms than to the general endometriosis population.

## 5 Conclusion

As the first meta‐analysis to evaluate low‐FODMAP dietary interventions in women with endometriosis, this study provides preliminary evidence of a potential signal of benefit, establishing diet as a relevant non‐pharmacological tool. However, these findings should be viewed strictly as hypothesis‐generating associations rather than definitive evidence of therapeutic efficacy. To move beyond these preliminary associations and inform future clinical guidelines, larger, well‐powered, and well‐designed randomised controlled trials are urgently required. Future trials should standardise FODMAP restriction with longer follow‐up, and actively stratify patients by baseline gastrointestinal symptom burden, IBS‐like phenotype, and intervention duration to confirm true clinical efficacy.

## Author Contributions

**Amanda Alves Rabelo:** conceptualisation, methodology, data curation, formal analysis, writing – original draft. **Janete Rabelo Rios:** conceptualisation, methodology, data curation, formal analysis, writing – original draft. **Júlia Fraga Castro e Oliveira:** validation, writing – original draft; review and editing. **Yasmim de Souza Nunes:** validation, writing – original draft, review and editing. **Gabriel Bernardes Yacoub:** conceptualisation, methodology, data curation. **Carolina Alves Corrêa:** validation, writing – original draft, review and editing. **Guilherme Santos da Silva:** writing – original draft, review and editing. **Alessandra Bedin‐Pochini:** resources, supervision, writing – review and editing, project administration.

## Funding

The authors have nothing to report.

## Disclosure

All authors report no relationships that could be construed as a conflict of interest. All authors take responsibility for all aspects of the reliability and freedom from bias of the data presented and their discussed interpretation.

## Ethics Statement

Not required for this study as it is a systematic review and meta‐analysis of previously published data.

## Conflicts of Interest

The authors declare no conflicts of interest.

## Supporting information


Supporting File

## Data Availability

The template data collection forms, data extracted from included studies, data used for all analyses, analytic code, and other materials used in this review are available from the corresponding author upon reasonable request.
