# Supplementary material for: Effects of a Low‐FODMAP Diet on Symptoms and Quality of Life in Women With Endometriosis: A Systematic Review and Meta‐Analysis
Source: J Hum Nutr Diet. 2026 Oct 14;39(5):e70353. doi: 10.1111/jhn.70353 (PMC13669521; doi:10.1111/jhn.70353)
Supplement: Supplementary file 1 [file JHN-39-0-s001.docx]

## **SUPPLEMENTARY APPENDIX**

**Summary**

Supplementary Table 1: Full search strategy	1

Supplementary Table 2. The PRISMA checklist	4

Supplementary Table 3 - Inclusion criteria of the included studies	7

Supplementary Table 4 - Exclusion criteria of the included studies	8

Supplementary Table 4 - Analysis of qualitative outcomes	9

Supplementary Table 5 –Newcastle-Ottawa Scale risk of bias	10

Supplementary Table 6 – RoB 2 risk of bias	10

Supplementary Figure 3 - Leave-one-out analysis	11

Supplementary Table 7 - GRADE assessment	12

### Supplementary Table 1: Full search strategy

| Database | Search Strategy |
| --- | --- |
| PubMed | ("endometriosis"[mh] OR endometriosis[tiab]) AND ("FODMAP Diet"[mh] OR fodmap*[tiab] OR "low FODMAP"[tiab] OR "low-FODMAP"[tiab] OR "FODMAPs"[tiab] OR "fermentable oligo-, di-, mono-saccharides and polyols"[tiab] OR "fermentable oligosaccharides, disaccharides, monosaccharides and polyols"[tiab] OR "Endometriosis/diet therapy"[mh] OR "Diet Therapy"[mh] OR diet*[tiab] OR dietary[tiab] OR "dietary intervention"[tiab] OR "dietary management"[tiab] OR "nutrition therapy"[tiab] OR "nutritional therapy"[tiab] OR "nutrition intervention"[tiab] OR "nutritional treatment"[tiab] OR "medical nutrition"[tiab]) |
| Embase | ('endometriosis'/exp OR 'endometriosis':ti,ab,kw) AND ('FODMAP diet'/exp OR 'fodmap*':ti,ab,kw OR 'low fodmap':ti,ab,kw OR 'low-fodmap':ti,ab,kw OR 'fodmaps':ti,ab,kw OR 'fermentable oligo-, di-,mono-saccharides and polyols':ti,ab,kw OR 'fermentable oligosaccharides, disaccharides, monosaccharides and polyols':ti,ab,kw OR 'endometriosis'/exp/dm_dm OR 'diet therapy'/exp OR 'diet*':ti,ab,kw OR 'dietary':ti,ab,kw OR 'dietary intervention':ti,ab,kw OR 'dietary management':ti,ab,kw OR 'nutrition therapy':ti,ab,kw OR 'nutritional therapy':ti,ab,kw OR 'nutrition intervention':ti,ab,kw OR 'nutritional treatment':ti,ab,kw OR 'medical nutrition':ti,ab,kw) |
| Cochrane | ("endometriosis") AND ("FODMAP diet" OR fodmap OR "low FODMAP" OR "fermentable oligosaccharides disaccharides monosaccharides and polyols" OR "Diet Therapy" OR diet OR "Nutrition Therapy" OR dietary OR "dietary intervention" OR "dietary management" OR "nutrition therapy" OR "nutritional therapy" OR "nutrition intervention" OR "nutritional treatment" OR "medical nutrition") |

### Supplementary Table 2. The PRISMA checklist

| **Section and Topic** | **Item #** | **Checklist item** | **Location where the item is reported** |
| --- | --- | --- | --- |
| **TITLE** | | |  |
| Title | 1 | Identify the report as a systematic review. | 1 |
| **ABSTRACT** | | |  |
| Abstract | 2 | See the PRISMA 2020 for Abstracts checklist. | 2 |
| **INTRODUCTION** | | |  |
| Rationale | 3 | Describe the rationale for the review in the context of existing knowledge. | 4 |
| Objectives | 4 | Provide an explicit statement of the objective(s) or question(s) the review addresses. | 5 |
| **METHODS** | | |  |
| Eligibility criteria | 5 | Specify the inclusion and exclusion criteria for the review and how studies were grouped for the syntheses. | 5-6 |
| Information sources | 6 | Specify all databases, registers, websites, organisations, reference lists, and other sources searched or consulted to identify studies. Specify the date when each source was last searched or consulted. | 6 |
| Search strategy | 7 | Present the full search strategies for all databases, registers, and websites, including any filters and limits used. | 2-3 (Supp. Material) |
| Selection process | 8 | Specify the methods used to decide whether a study met the inclusion criteria of the review, including how many reviewers screened each record and each report retrieved, whether they worked independently, and, if applicable, details of automation tools used in the process. | 5 |
| Data collection process | 9 | Specify the methods used to collect data from reports, including how many reviewers collected data from each report, whether they worked independently, any processes for obtaining or confirming data from study investigators, and, if applicable, details of automation tools used in the process. | 6 |
| Data items | **10a** | List and define all outcomes for which data were sought. Specify whether all results that were compatible with each outcome domain in each study were sought (e.g., for all measures, time points, analyses), and if not, the methods used to decide which results to collect. | 7 |
|  | **10b** | List and define all other variables for which data were sought (e.g., participant and intervention characteristics, funding sources). Describe any assumptions made about any missing or unclear information. | 5-6 |
| Study risk of bias assessment | 11 | Specify the methods used to assess risk of bias in the included studies, including details of the tool(s) used, how many reviewers assessed each study, and whether they worked independently, and if applicable, details of automation tools used in the process. | 7 |
| Effect measures | 12 | Specify for each outcome the effect measure(s) (e.g., risk ratio, mean difference) used in the synthesis or presentation of results. | 7-8 |
| Synthesis methods | **13a** | Describe the processes used to decide which studies were eligible for each synthesis (e.g,. tabulating the study intervention characteristics and comparing against the planned groups for each synthesis (item #5)). | 7 (Supp. Material) |
|  | 13b | Describe any methods required to prepare the data for presentation or synthesis, such as handling of missing summary statistics or data conversions. | 8 |
|  | 13c | Describe any methods used to tabulate or visually display the results of individual studies and syntheses. | 8 |
|  | 13d | Describe any methods used to synthesize results and provide a rationale for the choice(s). If meta-analysis was performed, describe the model(s), method(s) to identify the presence and extent of statistical heterogeneity, and software package(s) used. | 7-8 |
|  | 13e | Describe any methods used to explore possible causes of heterogeneity among study results (e.g., subgroup analysis, meta-regression). | 8 |
|  | 13f | Describe any sensitivity analyses conducted to assess the robustness of the synthesized results. | 8 |
| Reporting bias assessment | 14 | Describe any methods used to assess risk of bias due to missing results in a synthesis (arising from reporting biases). | 7 |
| Certainty assessment | 15 | Describe any methods used to assess certainty (or confidence) in the body of evidence for an outcome. | 8 |
| **RESULTS** | | |  |
| Study selection | 16a | Describe the results of the search and selection process, from the number of records identified in the search to the number of studies included in the review, ideally using a flow diagram. | 8 |
|  | 16b | Cite studies that might appear to meet the inclusion criteria, but which were excluded, and explain why they were excluded. | 9-10 |
| Study characteristics | 17 | Cite each included study and present its characteristics. | 9-10 |
| Risk of bias in studies | 18 | Present assessments of risk of bias for each included study. | 10 (Supp. Material) |
| Results of individual studies | 19 | For all outcomes, present, for each study: (a) summary statistics for each group (where appropriate) and (b) an effect estimate and its precision (e.g. confidence/credible interval), ideally using structured tables or plots. | 9-10 |
| Results of syntheses | 20a | For each synthesis, briefly summarise the characteristics and risk of bias among contributing studies. | 10-11 |
|  | **20b** | Present the results of all statistical syntheses conducted. If meta-analysis was done, present for each the summary estimate and its precision (e.g. confidence/credible interval) and measures of statistical heterogeneity. If comparing groups, describe the direction of the effect. | 10-11 |
|  | 20c | Present the results of all investigations of possible causes of heterogeneity among study results. | 12 |
|  | 20d | Present the results of all sensitivity analyses conducted to assess the robustness of the synthesized results. | 11-12 |
| Reporting biases | 21 | Present assessments of risk of bias due to missing results (arising from reporting biases) for each synthesis assessed. | 10-11 |
| Certainty of evidence | 22 | Present assessments of certainty (or confidence) in the body of evidence for each outcome assessed. | 12 (Supp. Material) |
| **DISCUSSION** | | |  |
| Discussion | 23a | Provide a general interpretation of the results in the context of other evidence. | 12-16 |
|  | 23b | Discuss any limitations of the evidence included in the review. | 15-16 |
|  | 23c | Discuss any limitations of the review processes used. | 15-16 |
|  | 23d | Discuss implications of the results for practice, policy, and future research. | 15-16 |
| **OTHER INFORMATION** | | |  |
| Registration and protocol | 24a | Provide registration information for the review, including register name and registration number, or state that the review was not registered. | 4 |
|  | 24b | Indicate where the review protocol can be accessed, or state that a protocol was not prepared. | 4 |
|  | 24c | Describe and explain any amendments to information provided at registration or in the protocol. | 4 |
| Support | 25 | Describe sources of financial or non-financial support for the review, and the role of the funders or sponsors in the review. | 16 |
| Competing interests | 26 | Declare any competing interests of review authors. | 16 |
| Availability of data, code and other materials | 27 | Report which of the following are publicly available and where they can be found: template data collection forms; data extracted from included studies; data used for all analyses; analytic code; any other materials used in the review. | 16-17 |

### Supplementary Table 3 - Inclusion criteria of the included studies

| **Study** | **Inclusion criteria** |
| --- | --- |
| Haaps et al. (2023) | Women diagnosed with endometriosis (confirmed by radiological imaging or laparoscopy), who were experiencing insufficient benefit from their current medical treatment, and who reported a pain score ≥3 cm on the Visual Analog Scale (VAS) for at least one of the following symptoms: dysmenorrhea, deep dyspareunia, and/or chronic pelvic pain. Additionally, participants had to be sufficiently fluent in Dutch or English |
| Keukens et al. (2025) | Women premenopausal and ≥18 years old, with an endometriosis diagnosis confirmed by physical examination, ultrasound, MRI, or laparoscopy. A key inclusion criterion was the presence of debilitating bowel symptoms such as abdominal pain, bloating, flatulence, constipation, diarrhea, gurgling, urgency, or nausea |
| Varney et al. (2025) | Women aged 18 years to menopause who were living in Melbourne and had an endometriosis diagnosis confirmed by laparoscopy and/or ultrasound. A critical inclusion requirement was the presence of poorly controlled gastrointestinal (GI) symptoms, defined by answering 'no' to the screening question about whether their bowel and gut symptoms were adequately controlled over the past week, and ultimately by scoring >30mm on the overall GI symptoms Visual Analog Scale (VAS) at baseline. |

**Abbreviations:** cm - centimeter; VAS - Visual Analog Scale; GI - Gastrointestinal; MRI - Magnetic Resonance Imaging.

### Supplementary Table 4 - Exclusion criteria of the included studies

| **Study** | **Exclusion criteria** |
| --- | --- |
| Haaps et al. (2023) | Women that undergone surgery in the previous 6 weeks or are scheduled for surgery in the following 6 weeks, those with an imminent change in hormonal therapy in the following 6 weeks, those who were pregnant or breastfeeding, and those with a diagnosis of malignancy, inflammatory bowel disease (IBD), and/or lactose intolerance. |
| Keukens et al. (2025) | Women postmenopausal or aged <18 years old, unable to speak, read, or write Dutch, or if who had gastrointestinal co-morbidities such as food allergy, Crohn's disease, Ulcerative Colitis, Celiac disease, or a diagnosis of Diabetes Mellitus. Other reasons for exclusion included currently following another diet, being currently pregnant, or having planned endometriosis surgery during the research period |
| Varney et al. (2025) | Women with irregular periods, laparoscopic surgery within the past 3 months, other GI disorders (such as Coeliac disease), food allergies and/or special diets, uncontrolled physical or mental illness, inability to read or understand English, being pregnant, planning pregnancy, or breastfeeding, having a surgical or medical contraindication, or being post-menopause. |

**Abbreviations**: IBD - Inflammatory Bowel Disease; GI - Gastrointestinal.

### Supplementary Table 4 - Analysis of qualitative outcomes

| **EHP-30 modular domain** | **Haaps et al.** | **Keukens et al.** | **Interpretation**  **(between-study comparison)** |
| --- | --- | --- | --- |
| **Work life** | 14.0 ± 16.9 | 26.2 ± 25.5 | Higher work-related impairment in the Keukens cohort (nearly double the mean score). |
| **Relationship with children** | NR | NR | Not available in the provided excerpt (the text under this heading is duplicated from “Sexual relationships”). |
| **Sexual relationships / sexual intercourse** | 30.3 ± 26.9 | 54.0 ± 31.0 | Largest divergence across domains; substantially higher impairment in Keukens. |
| **Interactions with healthcare professionals (medical profession)** | 17.5 ± 12.0 | 12.5 ± 43.8 | Higher scores in Haaps suggest greater perceived burden/less satisfaction in that cohort (as reported in the text). |
| **Treatment-related perceptions (treatment)** | 41.66 ± 22.9 | 41.7 ± 27.7 | Nearly identical mean scores, indicating comparable treatment-related perceptions. |
| **Infertility concerns (infertility)** | 56.3 (SD not reported) | 62.5 ± 43.8 | Highest absolute mean scores; infertility was a prominent concern in both cohorts, with higher burden in Keukens. |

### Supplementary Table 5 –Newcastle-Ottawa Scale risk of bias assessment for observational studies

| Study | Selection | | | | | Comparability | | | | Outcome | | | | Total score | Rating |
| --- | --- | --- | --- | --- | --- | --- | --- | --- | --- | --- | --- | --- | --- | --- | --- |
|  | Q1 | Q2 | Q3 | Q4 | T | Q1a | Q1b | T | Q1 | | Q2 | Q3 | T |  |  |
| Haaps, 2023 | * | * | * | - | 3 | - | - | 0 | - | | * | * | 2 | 5/9 | Fair |
| Keukens, 2025 | * | - | * | - | 2 | - | - | 0 | - | | * | - | 1 | 3/9 | Poor |

### Supplementary Table 6 – RoB 2 risk of bias assessment for randomized studies

| **Study** | **Bias from randomization process** | **Bias due to deviations from intended interventions** | **Bias due to missing outcome data** | **Bias in measurement of the outcomes** | **Bias in selection of the reported result** | **Overall risk of bias** |
| --- | --- | --- | --- | --- | --- | --- |
| **Varney, 2025** | Low risk | High risk | High risk | Low risk | Some concerns | High risk |

**Supplementary Figure 1** - Social support in patients with endometriosis.

**Supplementary Figure 2** - Self-image in patients with endometriosis.

### Supplementary Figure 3 - Leave-one-out sensitivity analysis of the pooled pain in patients with endometriosis consuming a low-FODMAP diet.

### Supplementary Table 7 - GRADE assessment

| **Outcome** | **Nº of studies** | **Categories** | | | | | **Effect** | | | **Certainly** |
| --- | --- | --- | --- | --- | --- | --- | --- | --- | --- | --- |
|  |  | **Risk of bias** | **Inconsistency** | **Indirectness** | **Imprecision** | **Publication Bias** | **MD** | **Nº of patients** | **Rate (95% CI)** |  |
| Pain | 3 | Serious¹ | Serious² | Not serious | Serious³ | Strongly suspected^4^ | 28.45 | 71 | 20.88 to 36.02 | Very Low  ⊕◯◯◯ |
| Control and helplessness | 3 | Serious¹ | Very serious^5^ | Not serious | Serious³ | Strongly suspected^4^ | 39.33 | 71 | 24.92 to 53.73 | Very Low  ⊕◯◯◯ |
| Emotional well-being | 3 | Serious¹ | Not serious | Not serious | Serious³ | Strongly suspected^4^ | 31.87 | 71 | 27.15 to 36.59 | Low  ⊕⊕◯◯ |
| Social support | 3 | Serious¹ | Not serious | Not serious | Serious³ | Strongly suspected^4^ | 38.94 | 71 | 33.69 to 44.20 | Low  ⊕⊕◯◯ |
| Self-image | 3 | Serious¹ | Not serious | Not serious | Serious³ | Strongly suspected^4^ | 43.22 | 71 | 35.94 to 50.49 | Low  ⊕⊕◯◯ |

*Abbreviations: MD, Mean Difference; Nº, number; CI, confidence interval.*

1. Serious risk of bias due to inclusion of two observational studies with moderate to high risk of confounding and one randomized controlled trial judged as high risk of bias because of deviations from intended interventions and missing outcome data.
2. Serious inconsistency due to substantial heterogeneity across studies (I² = 61.8%), likely reflecting differences in study design, baseline gastrointestinal symptom burden, and duration of dietary intervention.
3. Serious imprecision due to small sample size (n = 71 across three studies) and wide confidence intervals.
4. Publication bias is strongly suspected due to the small number of included studies and the absence of unpublished or negative trials.
5. Very serious inconsistency due to considerable heterogeneity (I² = 81.7%), indicating substantial variability in effect estimates across studies.
